# Supplementary material for: Wireless theranostic smart contact lens for monitoring and control of intraocular pressure in glaucoma
Source: Nat Commun. 2022 Nov 10;13:6801. doi: 10.1038/s41467-022-34597-8 (PMC9649789; doi:10.1038/s41467-022-34597-8)
Supplement: Supplementary file 1 — Supplementary Information [file 41467_2022_34597_MOESM1_ESM.pdf]

Supplementary Information

Wireless Theranostic Smart Contact Lens for Monitoring and Control of Intraocular Pressure in Glaucoma

Tae Yeon Kim[1], Jee Won Mok[2], Sang Hoon Hong[1], Sang Hoon Jeong[1], Hyunsik Choi[3], Sangbaie Shin[3], Choun-Ki Joo[2] and Sei Kwang Hahn[1,3,*]

[1] Department of Materials Science and Engineering, Pohang University of Science and Technology (POSTECH), 77 Cheongam-ro, Nam-gu, Pohang, Gyeongbuk 37673, Korea.

[2] CK St. Mary's Eye Center, CK building, 559, Gangnam-daero, Seocho-gu, Seoul 06531, Korea.

[3] PHI BIOMED Co., 168, Yeoksam-ro, Gangnam-gu, Seoul 06248, Korea.

*** CORRESPONDING AUTHOR FOOTNOTE**

Tel.: +82 54 279 2159; Fax: +82 54 279 2399; E-mail address: skhanb@postech.ac.kr (S. K. Hahn)

Supplementary Discussion

Hollow nanomaterials have been widely investigated for advanced applications with their unique properties. Especially, hollow gold nanomaterials have unique optical properties. The absorbance of hollow gold nanomaterials can be changed by plasmon hybridization[1]. This unique optical property of hollow gold nanomaterials has been used for various applications such as surface Raman enhanced spectroscopy (SERS)[2] and photothermal therapy (PTT)[3]. Remarkably, the hollow structure of nanomaterials can be easily deformed by mechanical stress. Some studies have reported the highly sensitive pressure sensor by using hollow nanomaterials[4]. We synthesized AuHNWs for long-term and accurate IOP monitoring taking advantages of the unique properties of hollow nanomaterials. The high sensitivity and stability of sensors are essential for long-term and accurate monitoring. Because AuHNW was prepared by using the Ag@AuNW, we used the Ag@AuNW as a control with the similar size and distribution (Supplementary Fig. 19). AuHNW was highly sensitive to strain, but relatively insensitive to temperature. Furthermore, we confirmed that the transmittance of AuHNW was higher than that of Ag@AuNW in the visible region due to the absorbance region shift. Highly sensitive, stable and transparent AuHNW would be a great nanomaterial for IOP sensing and other bioelectronic applications. Despite the FDA approval of Triggerfish, its commercial application was not successful possibly due to the low sensitivity of IOP sensor and the inconvenient accessories. In contrast, we greatly improved the sensitivity of IOP sensor using the AuHNW, and the wireless power and communication systems of our smart contact lens for further commercial application.

Diurnal-nocturnal intraocular pressure (IOP) fluctuation can be caused by diverse reasons such as posture change, cortisol levels, light changes and seasonal influences[5]. Eye blinking or rubbing can also cause the instantaneous IOP fluctuation. Although there is still no direct evidence, some

studies have revealed that these kinds of IOP fluctuations can affect the glaucoma progression[6-8]. Furthermore, it has been reported to be more efficient to observe glaucoma progression with continuous IOP monitoring for 24 h than IOP monitoring in a hospital over several visits[6-8]. In other words, it is important to continuously monitor IOP and collect many data for the analysis of the profile of IOP fluctuations, which can be effectively used to devise an appropriate management plan for glaucoma patients.

Our smart contact lens can be used to deliver drugs for the treatment of hypertension glaucoma model rabbits. Because the IOP of many glaucoma patients is higher than the normal IOP range, IOP is generally reduced by drug administration for the glaucoma management. Although the IOP of normal-tension glaucoma (NTG) is in the normal IOP range, the patients with NTG have been also highly recommended to maintain the low IOP[9]. The criteria of drug release from smart contact lens can be different in types of glaucoma patients and for the individual. It is necessary to set the criteria of drug release and develop the program for all types of glaucoma. However, our theranostic smart contact lens has high potential for further medical applications, because it would enable both the monitoring and control of IOP for the personalized therapy.

For treating glaucoma patients, it is still challenging to achieve high adherence of eye drop treatment[10]. In addition, because some IOP control drugs are known to cause ocular side effects after repeated topical drug delivery, it is critical to reduce the side effect of drugs for glaucoma treatment. The target IOP can be different for each patient in the different condition of glaucoma[11-13]. Accordingly, the exact dosage and the therapeutic plan should be determined by the discussion with ophthalmologists. Our smart contact lens would be greatly helpful for the ophthalmologists to determine the therapeutic plan by analyzing the individual data with long-term and continuous IOP monitoring. Our smart contact lens with a feedback system can deliver the appropriate amount of drugs in response to the IOP conditions. Taken together, our smart contact lens would maximize

the therapeutic effect and minimize the side effect of drugs by avoiding the unnecessary drug delivery for the next generation glaucoma treatment.

Supplementary Table 1. Types, characteristics and issues of smart contact lenses and drug eluting contact lenses with references.

Types	Characteristics	Issues	Ref.
IOP sensor lens (Triggerfish)	● FDA approved smart contact lens<br>● Full integration for wireless power and communication	● Low sensitivity<br>● No drug delivery	14
IOP sensor lens (Shen et al.)	● Highly sensitive IOP sensor using graphene nanosheets<br>● Highly transparent IOP sensor	● Incomplete integration<br>● No drug delivery<br>● No animal tests	15
IOP sensor lens (Park et al.)	● Strain simulation on contact lens<br>● Full integration for wireless power and communication<br>● Transparent and stretchable smart contact lens	● Low biocompatibility of silver nanowire<br>● No drug delivery	16
Drug eluting lens (Tang et al.)	● Dual delivery of latanoprost and timolol<br>● Sustained drug delivery using micelles	● No IOP sensing<br>● Uncontrolled drug delivery	17
Drug eluting lens (Ho et al.)	● Diamond-nanogel drug delivery system<br>● Controlled release by lysozyme	● No IOP sensing	18
Drug eluting lens (Shah et al.)	● Pharmacokinetic analysis of drugs released from hydrogel contact lens<br>● Sustained release of drugs	● No IOP sensing<br>● Uncontrolled drug delivery	19
Theranostic smart contact lens (This work)	● Theranostic system to simultaneously monitor and control IOP<br>● Full integration for wireless power and communication<br>● Highly sensitive, stable and biocompatible AuHNW sensor<br>● On-demand controlled drug delivery<br>● *In vivo* glaucoma management		

Supplementary Methods

Synthesis of AgNW and Ag@AuNW

Silver nanowire was synthesized as a template by the slightly modified polyol method. In brief, EG (65 ml, 324558, Sigma Aldrich) was heated at 175 ^{o}C for 1 h with 0.034 M of PVP (Mw, 360000, Sigma Aldrich). After heating, 400 μl of 4 mM $CuCl_2$ (751944, Sigma Aldrich) was added and heated for 10 min. Then, 15 ml of 0.095 M $AgNO_3$ (209139, Sigma Aldrich) was injected with a syringe pump for 10 min. The reaction was conducted for 20 min and the resulting AgNW was washed multiple times with centrifugation. Ag@AuNW was synthesized by non-galvanic growth of Au on the silver nanowire. The growth solution was prepared by mixing 1.4 ml of 0.2 M hydrogen tetrachloroaurate (III) hydrate ($HAuCl_4{\cdot}xH_2O$) (Alfa Aesar), 105 ml of 0.01 M Na_2SO_3 (Sigma Aldrich) in 165 ml of deionized (DI) water and left for 12 h. After 12 h, the separation solution was prepared by mixing 70 ml of 50 wt% PVP (Mw; 40000, Sigma Aldrich), 14 ml of 0.5 M NaOH (757527, Sigma Aldrich), 14 ml of 0.5 M L-ascorbic acid (L-AA, A0278, Sigma Aldrich) and 3.5 ml of 0.1 M sodium sulfite (Na_2SO_3, S0505, Sigma Aldrich) in 320 ml DI water. The growth solution was added into the separation solution and the pH of the mixed solution was adjusted by adding 0.2 M NaOH. When the pH was saturated at 10, the silver nanowire solution with a different concentration was added into the mixed solution. The reaction was carried out for 2 h and the synthesized Ag@AuNW was washed with ethanol multiple times.

Preparation of glaucoma induced rabbits

Glaucoma was induced in both eyes of each animal under sterile condition. Rabbits were anaesthetized with ketamine (50 mg/kg) and Rompun (10 mg/kg) administered intramuscularly. Then, 0.5% alkyne eye drops were applied by topical instillation. After anesthetization of the

animals, 2% methyl cellulose (M0512, viscosity of 4,000cP, Sigma Aldrich) was injected into the anterior chamber with a 29-gauge needle of an insulin syringe and 50 U α-chymotrypsin (from bovine pancreas, C4129, Sigma Aldrich) into the posterior chamber[20]. Both eyes before each IOP measurement were anesthetized by one drop of 0.5 % alkyne. The first measurement was taken before IOP induction and the second was taken after 1 h. After that, IOP measurements were repeated with a tonometer twice a day, and an average of five IOP readings was used for the analysis. IOP measurements were performed before and after treatment with topical eye drops or drug delivery by wearing the smart contact lens. Glaucoma therapeutic eye drop (0.5% Timoptic, Bausch Lomb) into the right eye and saline instilled into the left eye were performed twice a day.

Corneal Fluorescein Staining

The cornea was stained with fluorescein paper strips (0.4M disodium fluorescein, HAAG-STREI DIAGNOSITECS) soaked in a drop of 0.5 % alkyne. After staining, it was washed with saline solution. The eyes of all animals were examined under a slit-lamp microscope (Keeler, UK) with a cobalt blue filter[21].

Histopathologic and Immunohistochemical Analyses

Formalin-fixed whole eyes were embedded in paraffin for the preparation of 5 μm sections. For histological evaluation, the sectioned tissues were stained with hematoxylin and eosin (H & E; ABCAM, UK) and examined under a direct light microscope. Briefly, immunohistochemical detection included antigen retrieval in 10 mM citrate buffer in a microwave oven and blocking endogenous peroxidase with 1% hydrogen peroxide. Tissues were incubated at 4 °C overnight with the following primary antibodies; GFAP (sc-51908, Santa Cruz), CD11b (ab8878, ABCAM), BDNF (ab108619, ABCAM) and Brn3a (ab345230, ABCAM). Then, the VECTASTAIN Elite

ABC reagent (horse anti-mouse/rabbit IgG, Vector Laboratories, Burlingame, CA) for horseradish peroxidase was used for immunohistochemistry. After that, tissues were incubated and stained in a peroxidase substrate solution (ImmPACT DAB Substrate, Peroxidase, Vector Laboratories, Burlingame, CA) up to the desired intensity, and lightly counterstained with nuclear fast red (Abcam, UK).

Calibration of IOP level (mmHg) measured by smart contact lens

To obtain the standard graph of each rabbit to convert code change to IOP level, the base codes of smart contact lens were measured before wearing smart contact lens without any tension (0 mmHg, C_0). The IOP level of each rabbit was measured by tonometer (χ mmHg). Right after that, the code outputs were measured by wearing smart contact lens (χ mmHg, C). The code changes obtained by equation (1) were matched with IOP changes with more than 2 points. The slope of standard graph of each rabbit was used to convert code changes (%) to IOP changes (mmHg) for each rabbit using equation (2).

$$\Delta C = Code\ change\ (\%) = \frac{(C - C_0)}{C_0} * 100 \qquad (1)$$

$$\Delta P = IOP\ change\ (mmHg) = \frac{\Delta C}{S} \qquad (2)$$

$$S = Slope\ of\ standard\ graph\ of\ each\ rabbit$$

Before wearing contact lens, the initial IOP was measured by tonometer (P_0). The IOP changes converted by equation (2) were measured with smart contact lens and the IOP level which was desired to measure was calculated by equation (3). Finally, the IOP level measured by smart contact lens was compared to IOP level measured by tonometer right after remove the contact lens.

$$P = P_0 + \frac{\Delta C}{S} = P_0 + \Delta P \qquad (3)$$

Thermal analyses of theranostic smart contact lens

The thermal characterization of theranostic smart contact lens was carried out with the infrared camera for the temperature changes of IOP sensor, antenna, chip and DDS during *in vivo* tests. Although the temperature of chip increased up to 39.3 °C, there were not any critical thermal damages on the cornea after finishing all *in vivo* tests.

Assessment of ASIC Chip

The ASIC chip was fabricated using a 180 nm complementary metal-oxide semiconductor (CMOS) process in a die area of 2.25 mm^2 (Supplementary Fig. 12). The thickness of ASIC chip was ca. 200 μm. The IOP sensor and DDS were integrated with slightly modified ASIC chip[22]. The power management unit (PMU) could receive inductively-coupled wireless AC power and convert into DC power with CMOS rectifier. A potentiostat with three nodes of working electrode (WE), reference electrode (RE) and counter electrode (CE) was integrated into the ASIC chip. For integration of the IOP sensor with the ASIC chip, one electrode was connected to the integrated CE and RE, and the other electrode was connected to the WE. The applied voltage to IOP sensor was about 0.65 V. The sensed current of IOP sensor was converted to 15-bit digital output code by an analog-to-digital convertal (ADC) with the conversion range of ca. 43.7 μA and a resolution of 1.64 nA. The output data was transmitted through the antenna and synchronized with an on-chip clock generated by a relaxation oscillator. The wireless board (external reader) demodulated the amplitude-shift keying (ASK)-modulated RF signal to recover the transmitted packet of output data. The microcontroller (MCU) in the wireless board controlled the entire reader system and a

commercial Bluetooth module (BLE) for interface of external personal computer (PC). For the drug delivery system, anode and cathode were selectively connected to PMU. Each drug well could be selectively activated by the control commands received from the external reader.

Statistical analysis

We performed one-sided statistical analyses using one-way analysis of variance (ANOVA). For all experiments, $*P < 0.05$, $**P < 0.01$ and $***P < 0.001$ were considered statistically significant. All error bars represent the standard deviation.

Supplementary References

1. Prodan, E. et al. A hybridization model for the plasmon response of complex nanostructures. *Science* **302,** 419-422 (2003).
2. Kang, H. et al. Near-infrared SERS nanoprobes with plasmonic Au/Ag hollow-shell assemblies for in vivo multiplex detection. *Adv. Funct. Mater.* **23,** 3719-3727 (2013).
3. Lee, J. et al. Targeted hyaluronate-hollow gold nanosphere conjugate for anti-obesity photothermal lipolysis. *ACS Biomater. Sci. Eng.* **3,** 3646-3653 (2017).
4. Pan, L. et al. An ultra-sensitive resistive pressure sensor based on hollow-sphere microstructure induced elasticity in conducting polymer film. *Nat. Commun.* **5,** 1-8 (2014).
5. Kim, J. H. & Caprioli, J. Intraocular pressure fluctuation: is it important? *J. Ophthalmoic Vis. Res.* **13,** 170-174 (2018).
6. Kim, S. H. et al. The effect of diurnal fluctuation in intraocular pressure on the evaluation of risk factors of progression in normal tension glaucoma. *PLoS One* **11** (2016).

7. Renard, E. et al. Twenty-four hour (nyctohemeral) rhythm of intraocular pressure and ocular perfusion pressure in normal-tension glaucoma. *Invetig. Ophthalomol. Vis. Sci.* **51,** 882-889 (2010).

8. De Moraes C. G. et al. Visual field change and 24-hour IOP-related profile with a contact lens sensor in treated glaucoma patients. *Ophthalmology* **123,** 744-753 (2016).

9. Group, C. N. T. G. S. The effectiveness of intraocular pressure reduction in the treatment of normal-tension glaucoma. *Am. J. Ophthalmol.* **126,** 495-505 (1998).

10. Nordstrom, B. L. et al. Persistance and adherence with topical glaucoma therapy. *Am. J. Ophthalmol.* **140,** 598 (2005).

11. Sihota, R. et al. Simplifying "target" intraocular pressure for different stages of primary open-angle glaucoma and primary angle-closure glaucoma. *Indian J. Ophthalmol.* **66,** 495-505 (2018).

12. Hedman, K. and Larsson, L-. I. The effect of latanoprost compared with timolol in African-American, Asian, Gaucasian, and Mexican open-angle glaucoma or ocular hypertensive patients. *Surv. Ophthalmol.* **47,** S77-S89 (2002).

13. Heel, R. C. et al. Timolol: A reiveiw of its therapeutic efficacy in the topical treatment of glaucoma. *Drugs* **17,** 38-55 (1979).

14. Leonardi, et al. Wireless contact lens sensor for intraocular pressure monitoring: assessment on enucleated pig eyes. Acta Ophthalmol. 87, 433-437 (2009).

15. Liu, Z. et al. An ultrasensitive contact lens sensor based on self-assembly graphene for continuous intraocular pressure monitoring. *Adv. Funct. Mater.* **31**, 2010991 (2021).

16. Kim, J. et al. A soft and transparent contact lens for the wireless quantitative monitoring of intraocular pressure. *Nat. Biomed. Eng.* **5,** 772-782 (2021).

17. Xu, J. et al. Co-delivery of latanoprost and timolol from micelles-laden contact lenses for the treatment of glaucoma. *J. Control. Release* **305,** 18-28 (2019).

18. Kim, H. J., Zhang, K., Moore, L. & Ho, D. Diamond nanogel-embedded contact lenses mediate lysozyme-dependent therapeutic release. *ACS nano* **8**, 2998-3005 (2014).

19. Maulvi, F. A. et al. In vitro and in vivo evaluation of novel implantation technology in hydrogel contact lenses for controlled drug delivery. *J. Control. Release* **226,** 47-59 (2016).

20. Zhu, M. D. & Cai, F. Y. Development of experimental chronic intraocular hypertension in the rabbit. *J Ophthalmol*. **20,** 225-34 (1992).

21. Bron, A. J. et al. Grading of corneal and conjunctival staining in the context of other dry eye tests. *Cornea* **22**, 640–650 (2003).

22. Kim, S-. K. et al. Bimetallic nanocatalysts immobilized in nanoporous hydrogels for long-term robust continuous glucose monitoring of smart contact lens. *Adv. Mater.* **34,** 2110536 (2022).

Supplementary Figures

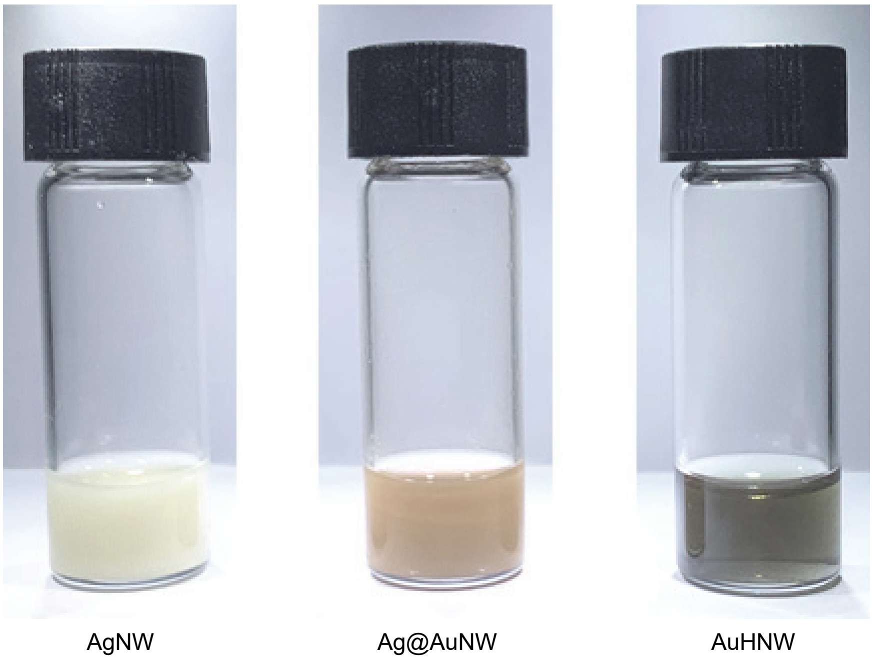

Supplementary Figure 1. Photo-images of the nanowire solutions of AgNW, Ag@AuNW and AuHNW.

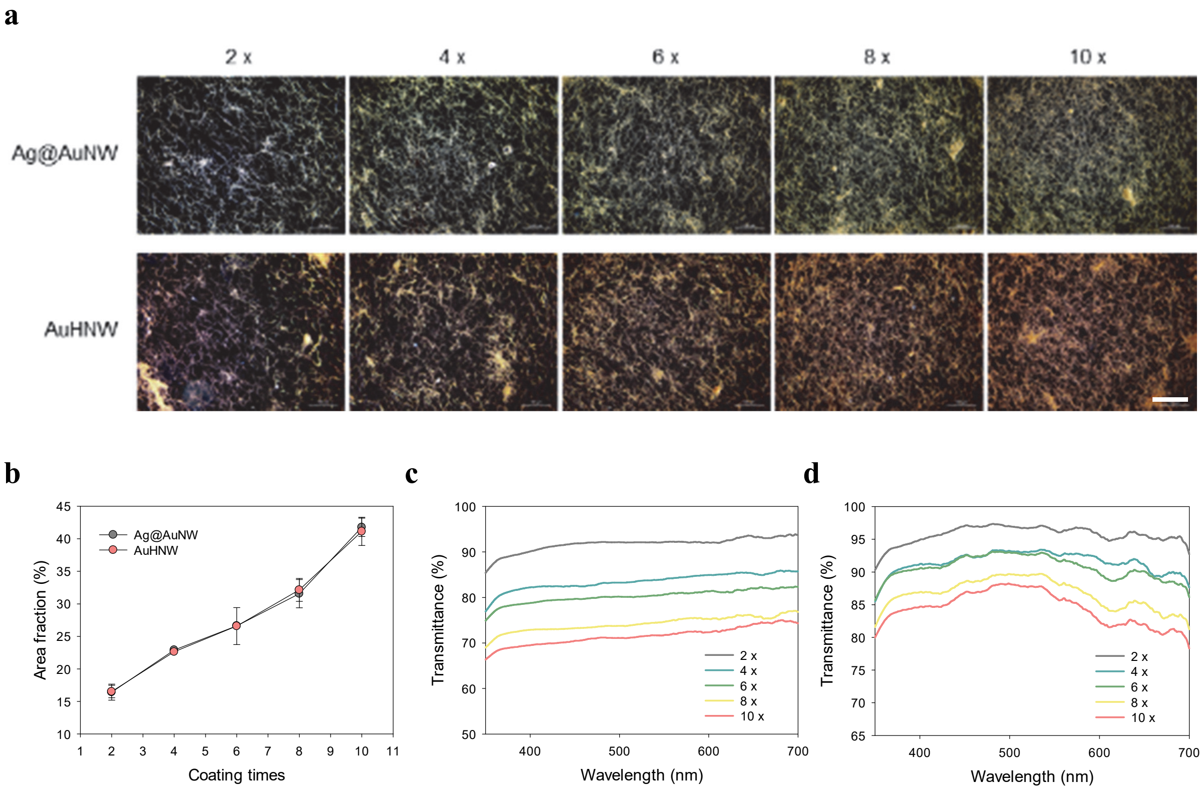

Supplementary Figure 2. The area fraction and transmittance of Ag@AuNW and AuHNW. **a,** OM dark field images of nanowire films with increasing coating times (scale bar, 50 µm). **b,** The area fraction of nanowire films with increasing coating times (n = 3, data are presented as a mean value ± SD). The transmittance spectrum of **c,** Ag@AuNW and **d,** AuHNW films with increasing coating times.

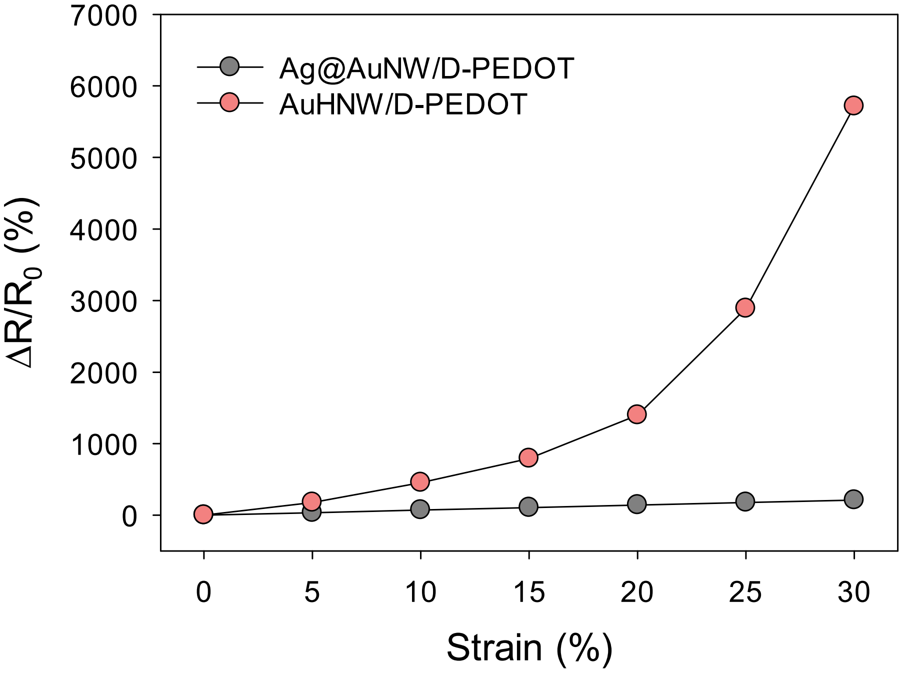

Supplementary Figure 3. The electromechanical characterization of hybrid films. The sensitivity of AuHNW/D-PEDOT was higher than that of Ag@AuNW/D-PEDOT.

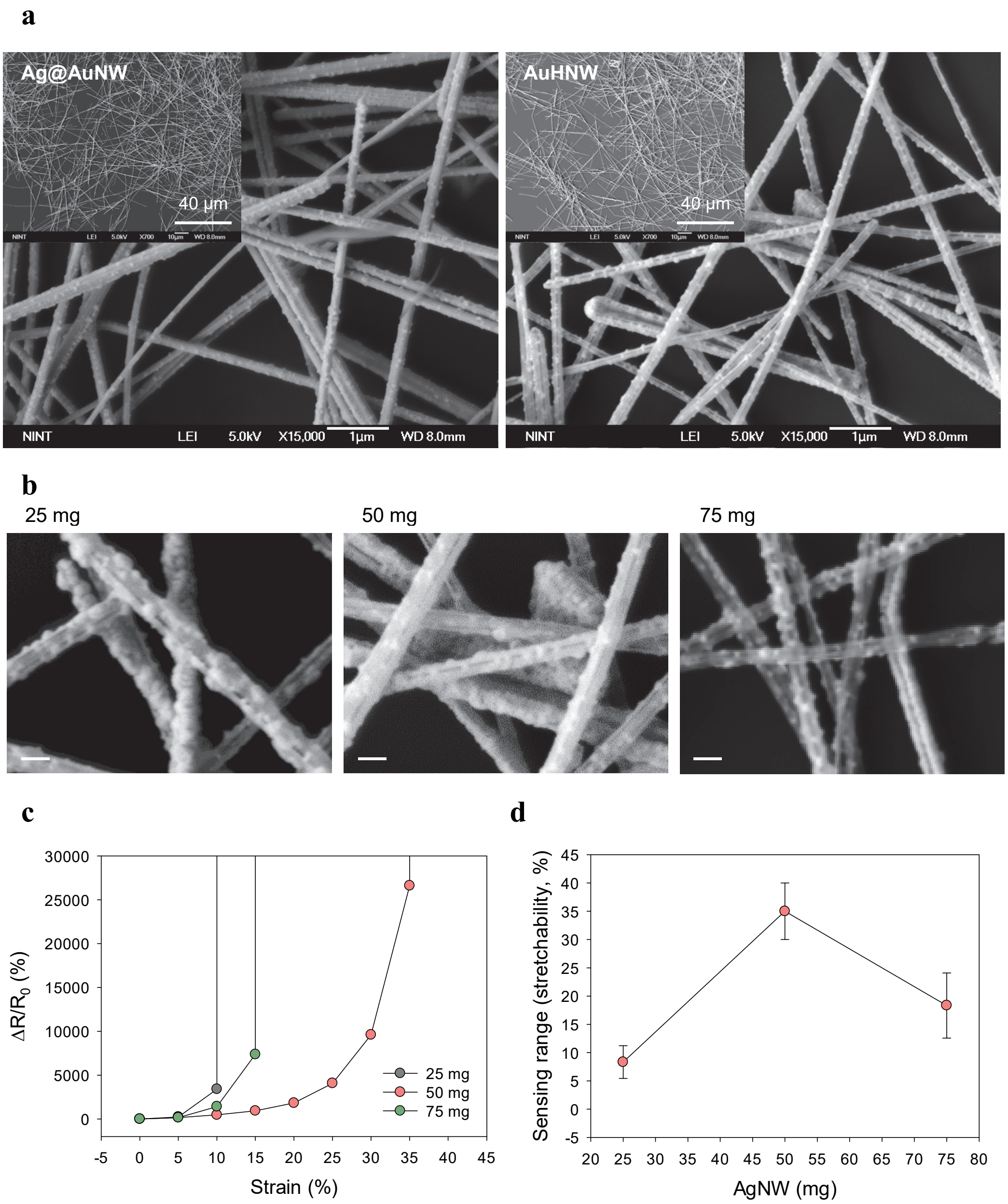

Supplementary Figure 4. SEM images and electromechanical properties of AuHNWs. a, SEM images of Ag@AuNW and AuHNW. **b,** SEM images of AuHNW with different template concentrations for tunning the shell thickness (scale bar, 200 nm). **c,** The relative resistance changes of AuHNW with different template concentrations. **d,** The sensing range of AuHNW with increasing concentration of AgNW templates (n = 3, data are presented as a mean value ± SD).

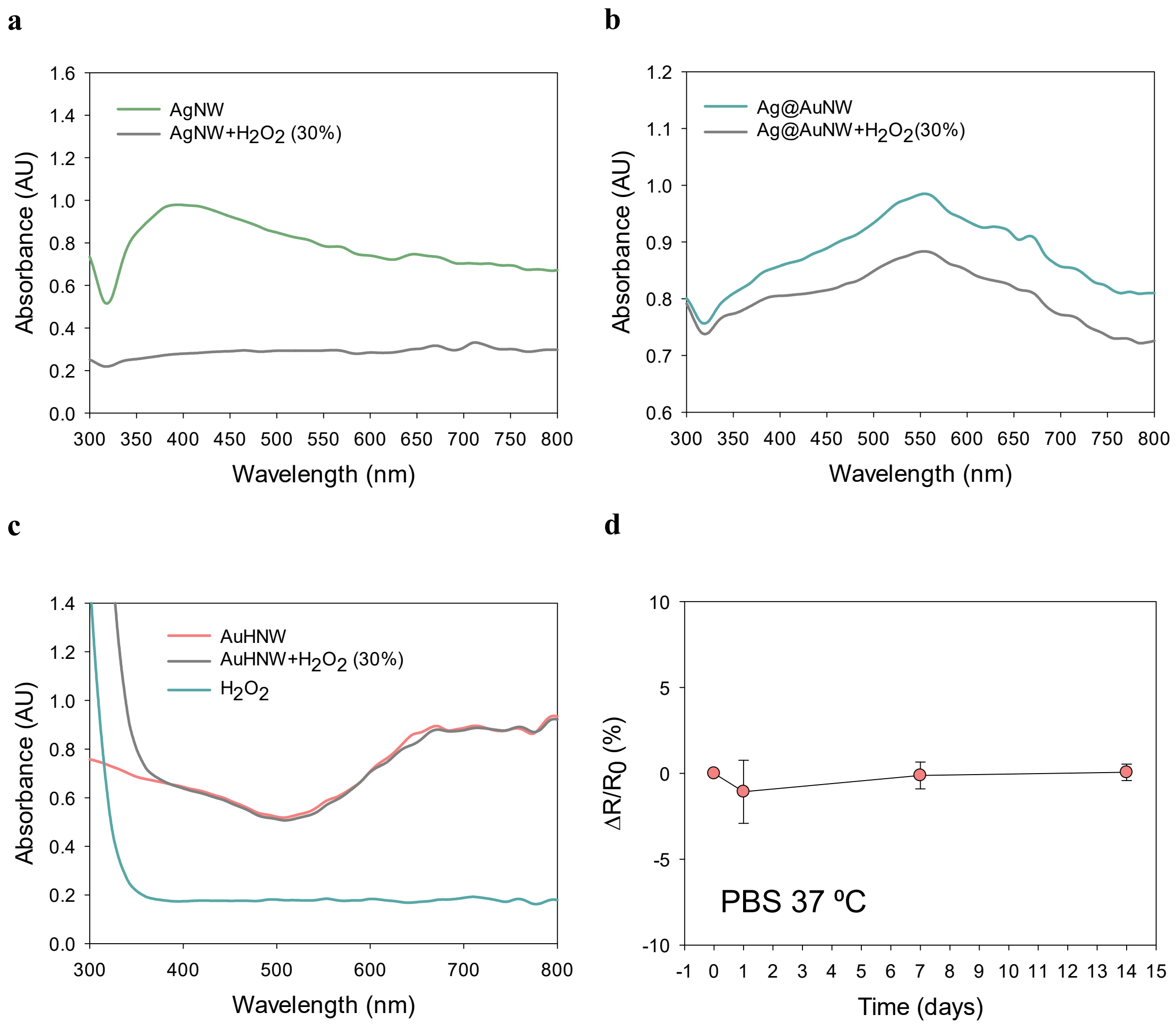

Supplementary Figure 5. Hydrogen peroxide exposure tests to assess the chemical stability. The absorbance change of **a,** AgNW, **b,** Ag@AuNW and **c,** AuHNW after exposure to H_2O_2 (30 %) for 2 h. **d,** The relative resistance change of AuHNWs in PBS at 37 °C (n = 3, data are presented as a mean value ± SD).

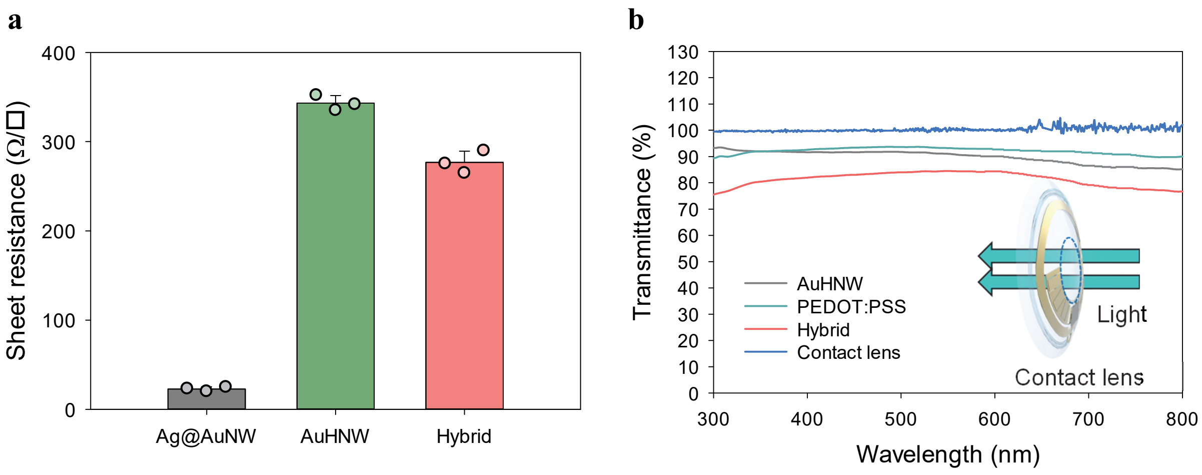

Supplementary Figure 6. Electrical and optical properties of hybrid IOP sensors. a, The sheet resistance (n = 3, data are presented as a mean value ± SD) and **b,** the transmittance of specified nanomaterials.

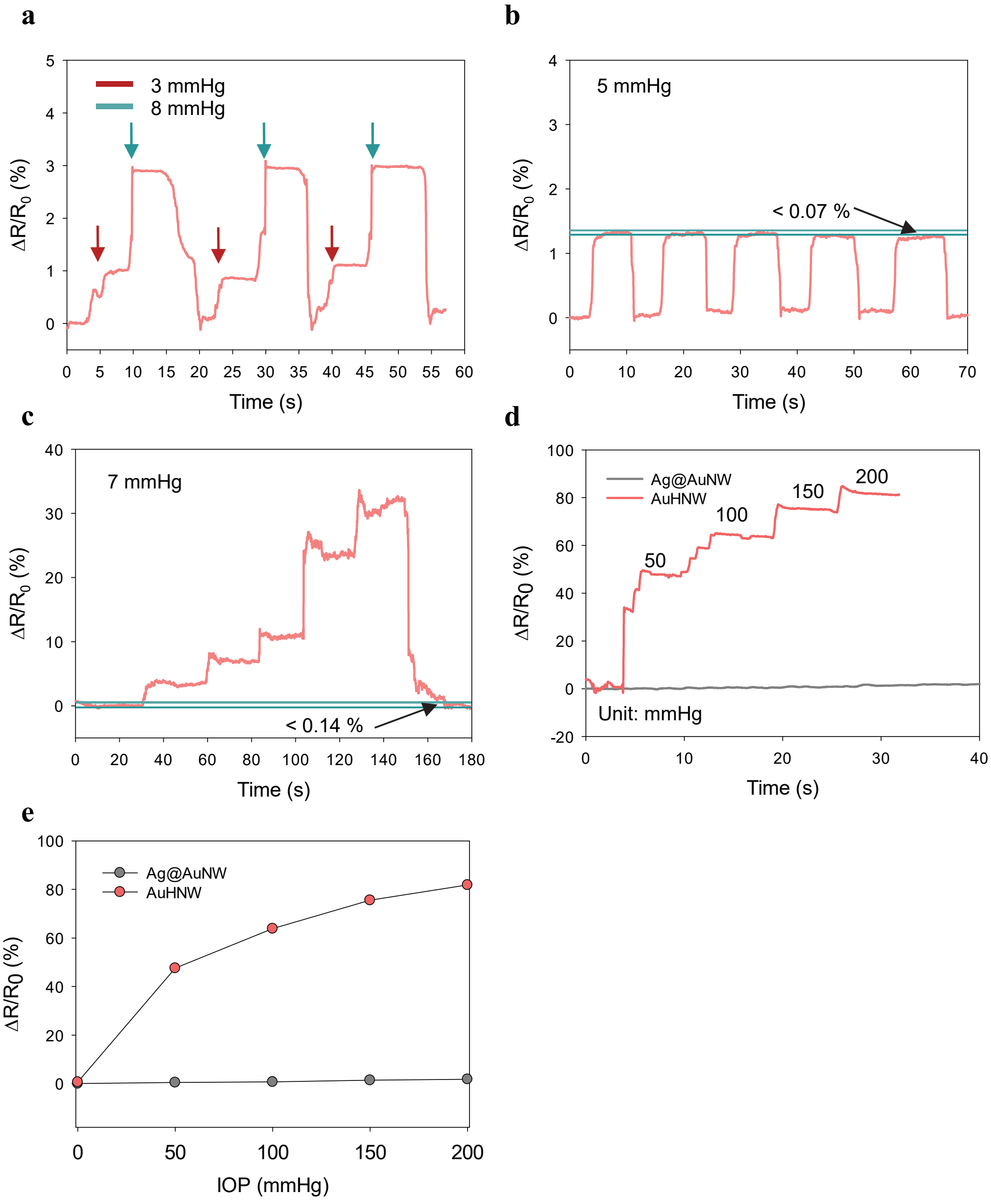

Supplementary Figure 7. Characteristics of the IOP sensor embedded into smart contact lens. **a,** The real time relative resistance change under the different applied IOP change. **b,** The repeated IOP change. **c,** The hysteresis of IOP sensor by applying IOP up to 35 mmHg and recovering to 0 mmHg. **d,** The real-time continuous monitoring and **e,** the relative resistance of Ag@AuNW and AuHNW IOP sensors in a wide range of IOP.

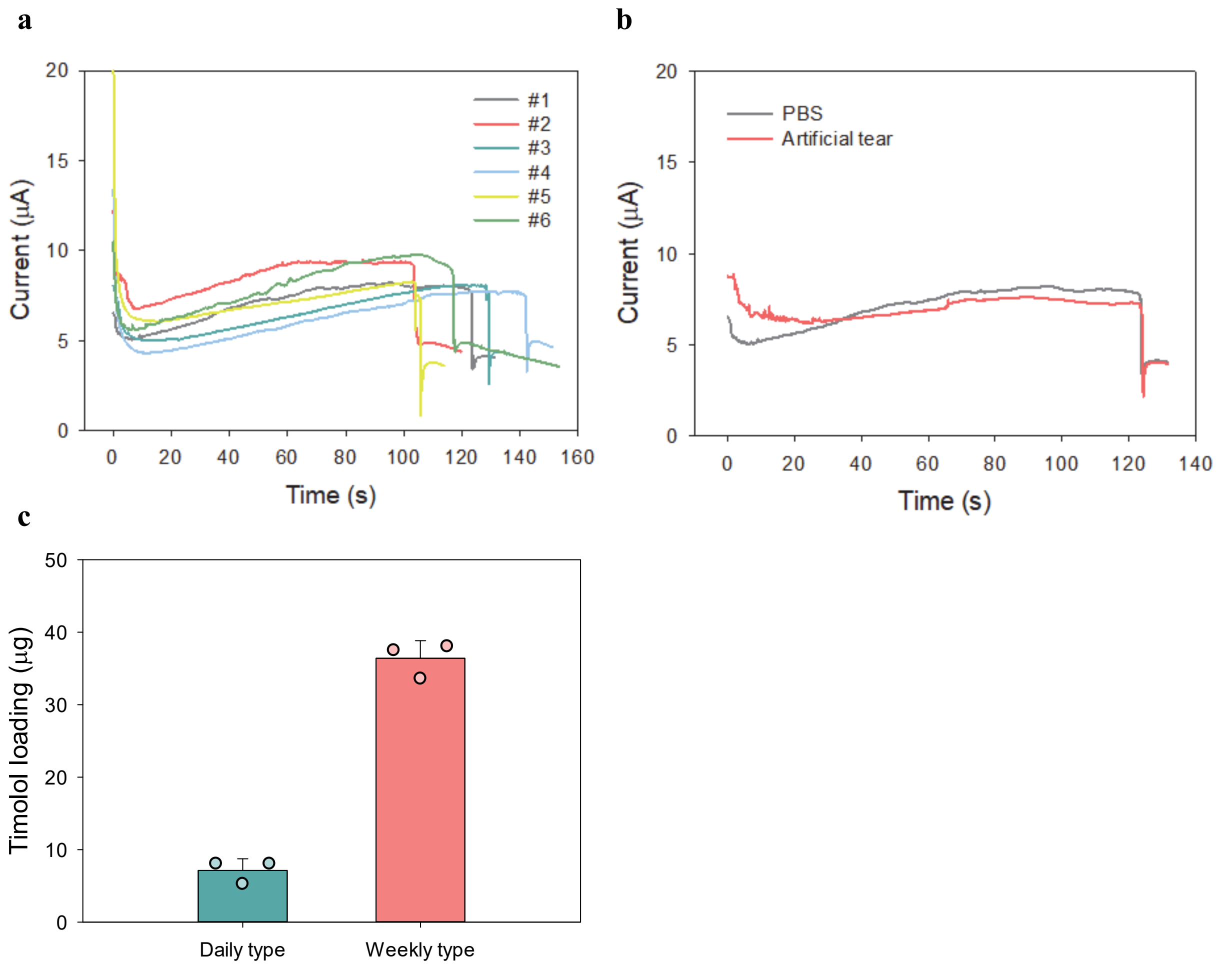

Supplementary Figure 8. Characteristics of flexible DDS. a, The current-time (I-t) curve of each reservoir of flexible DDS in pH 7.4 PBS with applying a constant voltage of 1.85 V. **b,** The electrochemical dissolution of DDS in PBS and artificial tear. **c,** The timolol loading amount for the different type of DDS (n = 3, data are presented as a mean value ± SD).

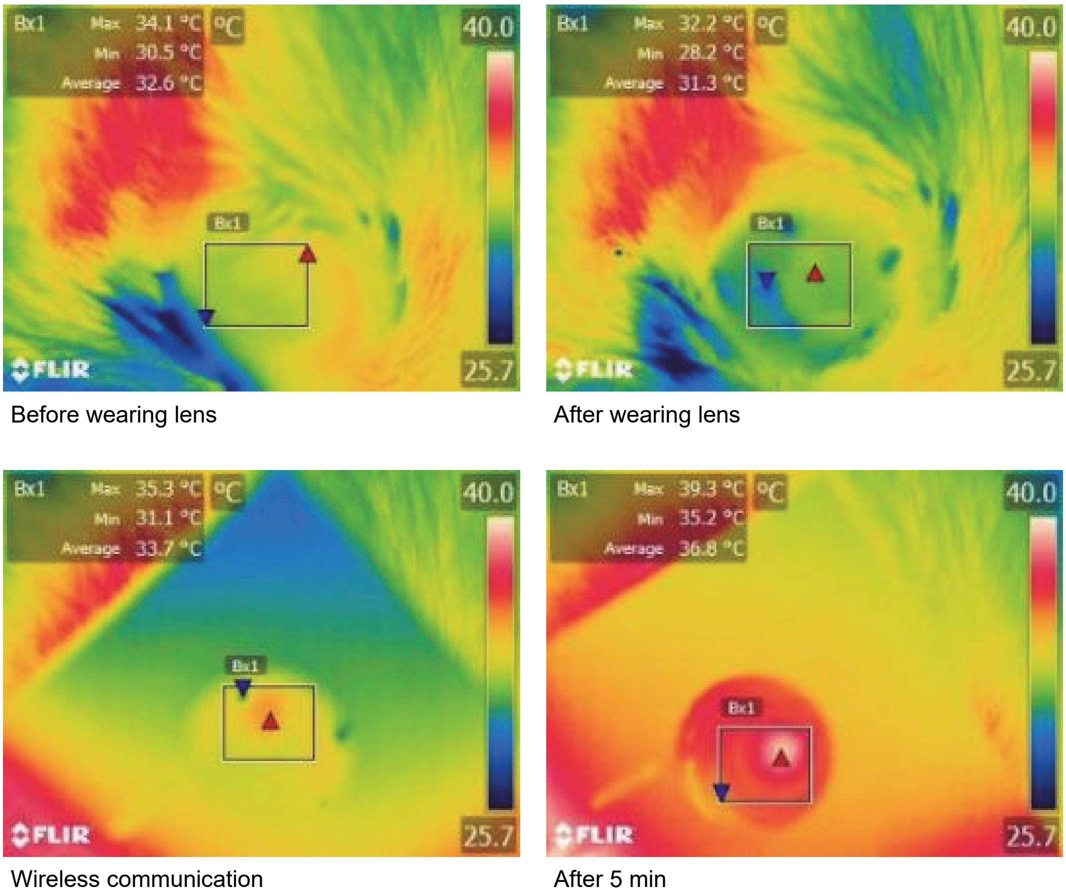

Supplementary Figure 9. The thermal characterization of theranostic smart contact lens.

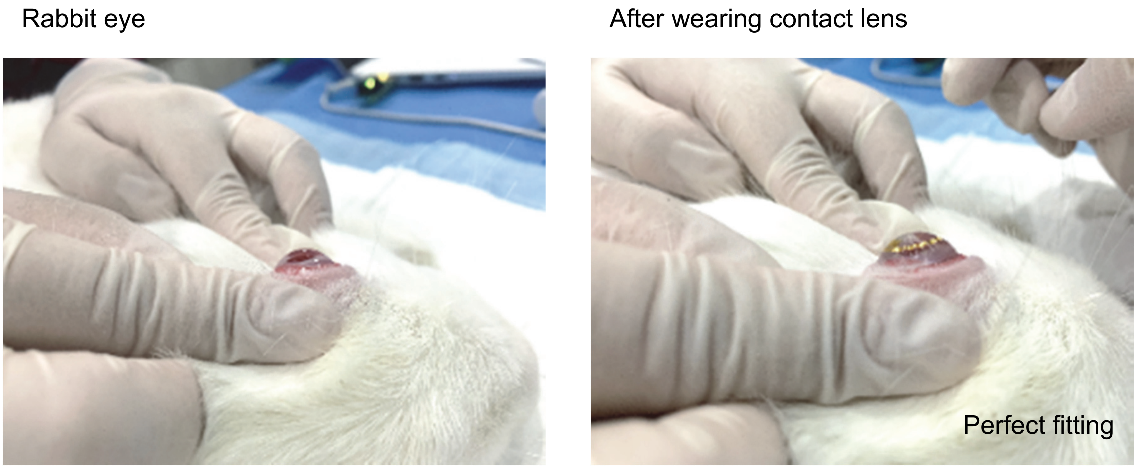

Supplementary Figure 10. The perfect fitting of theranostic smart contact lens on the rabbit eye. If a smart contact lens is not perfectly fit, there is a gap between the contact lens and the eye.

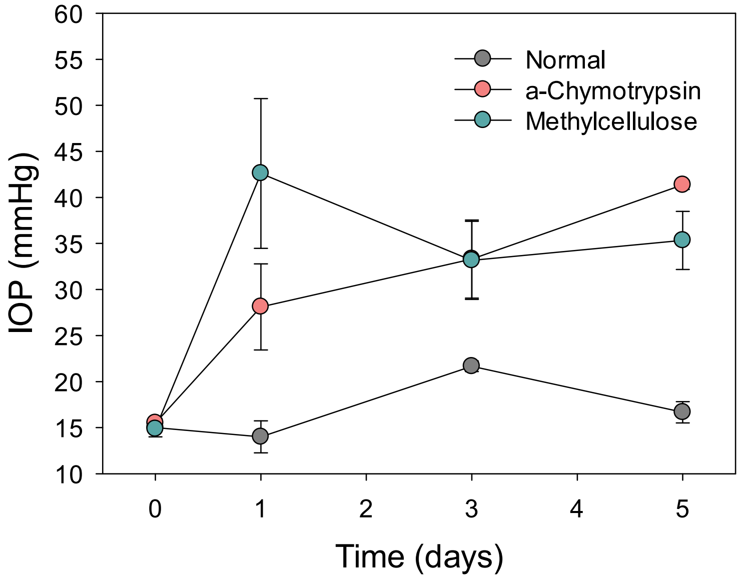

Supplementary Figure 11. The IOP value change of glaucoma induced rabbits by a tonometer (n = 3, data are presented as a mean value ± SD).

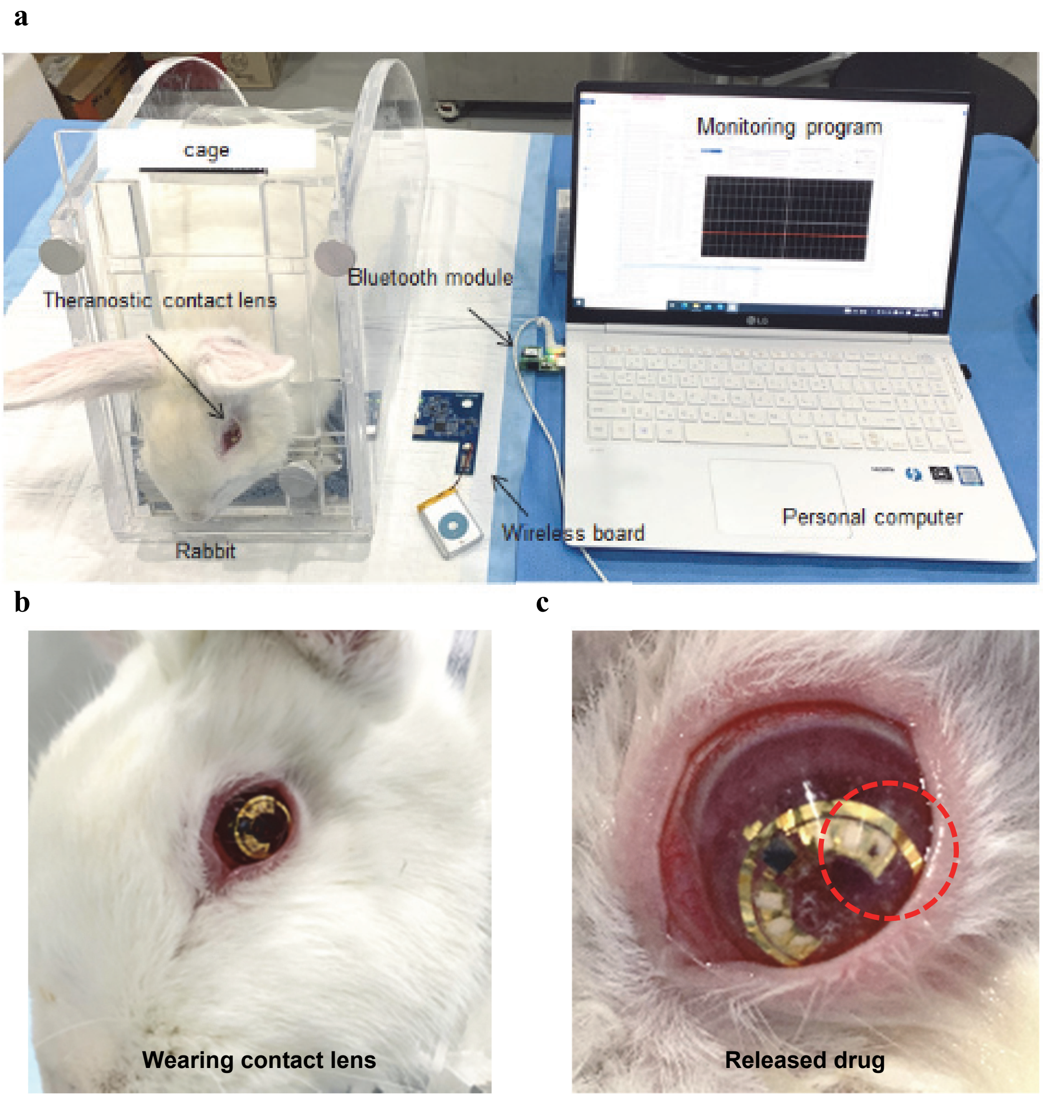

Supplementary Figure 12. ***In vivo*** **test set up with wireless theranostic smart contact lens.** **a,** *In vivo* test set up of theranostic smart contact lens for IOP sensing and drug delivery. Photo-image of rabbits **b,** wearing the smart contact lens, and **c,** smart contact lens on a rabbit eye after drug release.

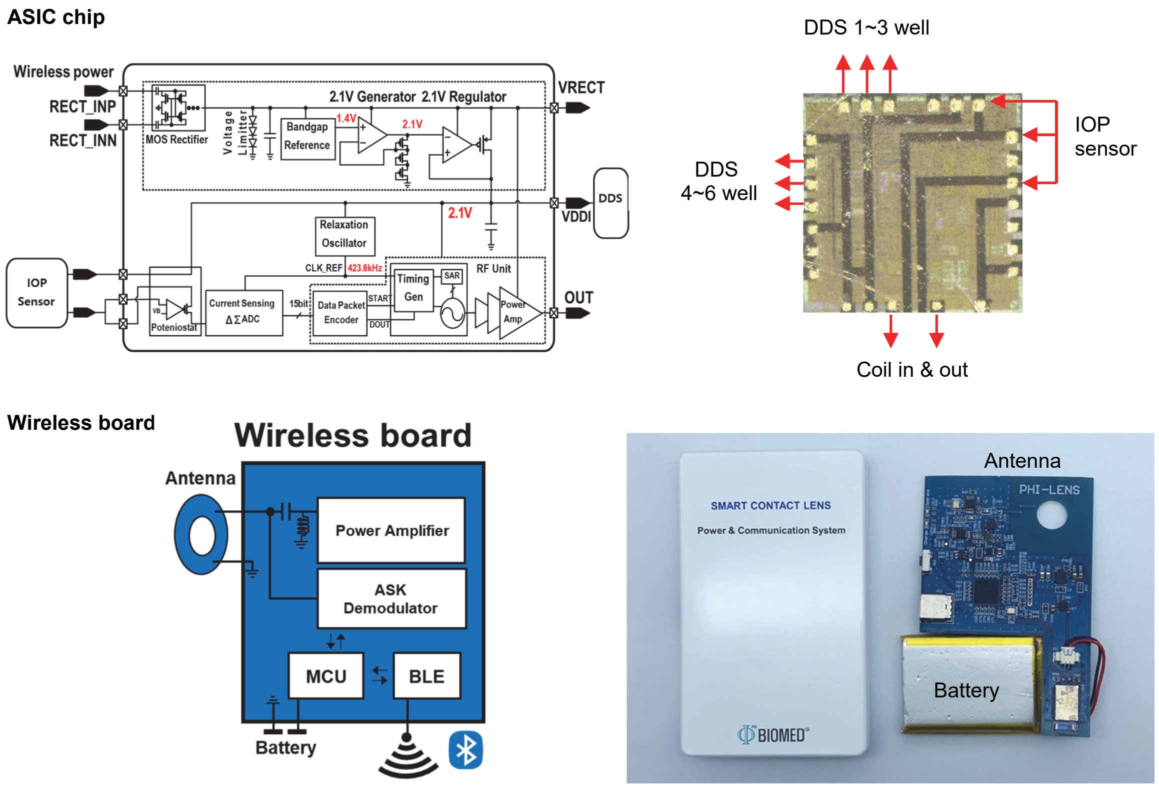

Supplementary Figure 13. Schematic illustration of an ASIC chip and a wireless power board for wireless power transmission and communication.

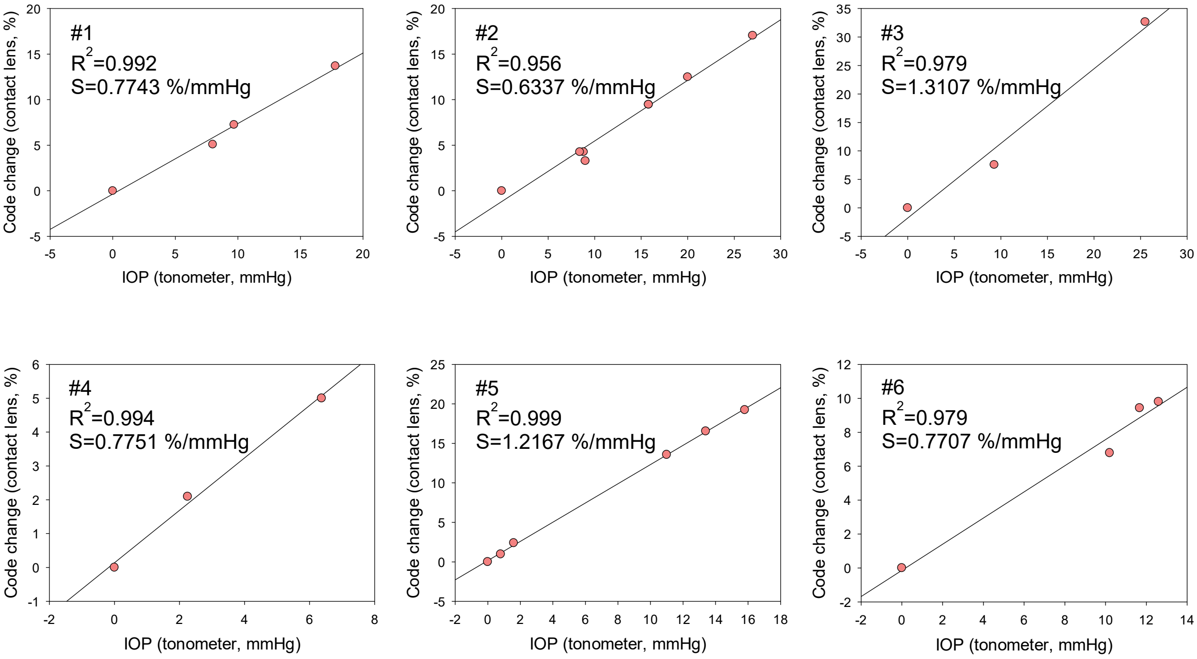

Supplementary Figure 14. The correlation curve between code changes and IOP values of glaucoma induced rabbits. These standard graphs were used for the calibration of smart contact lens for the code change into the IOP value.

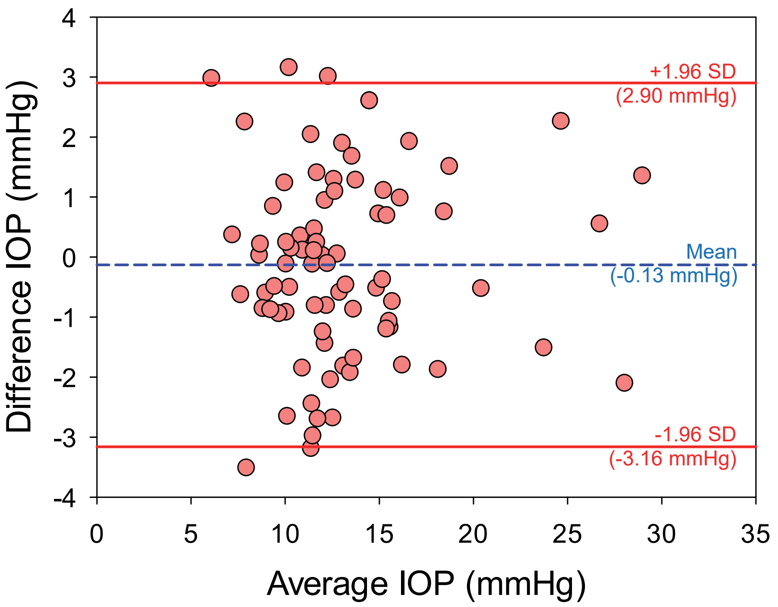

Supplementary Figure 15. Bland-Altman plot between a smart contact lens and a tonometer.

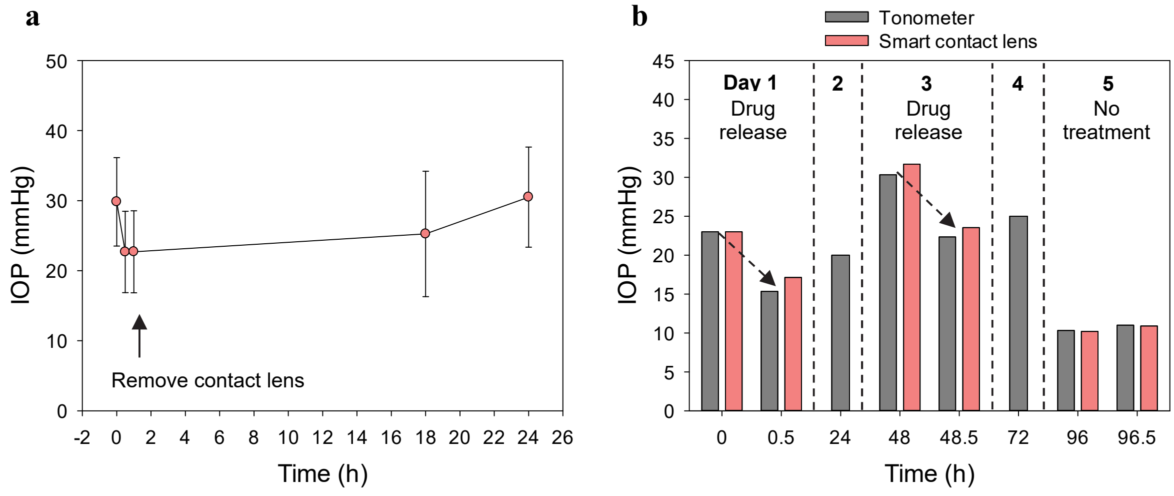

Supplementary Figure 16. The IOP change of glaucoma induced rabbits by timolol release. **a,** IOP changes after drug release for 24 h (n = 4, data are presented as a mean value ± SD). The IOP was measured with a tonometer. **b,** Daily IOP changes by simultaneous IOP monitoring and control for 5 days in Fig. 6e.

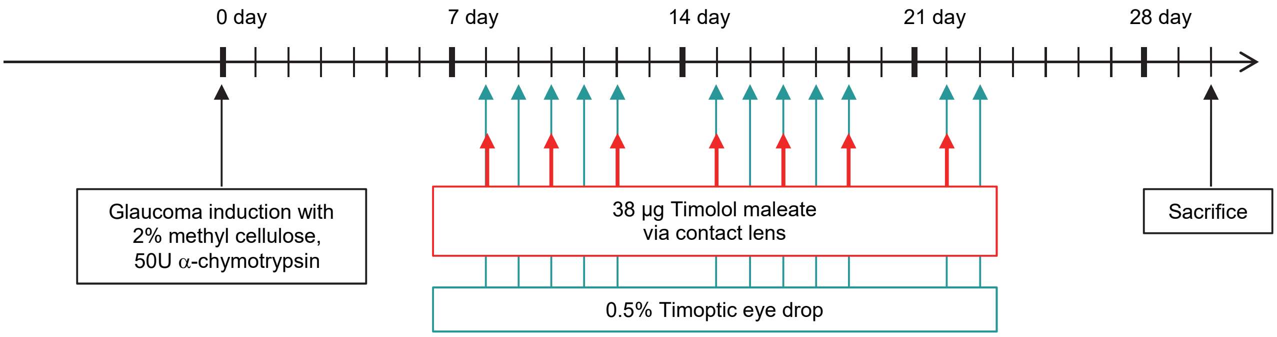

Supplementary Figure 17. In vivo test protocol for the assessment of therapeutic effects of theranostic smart contact lens.

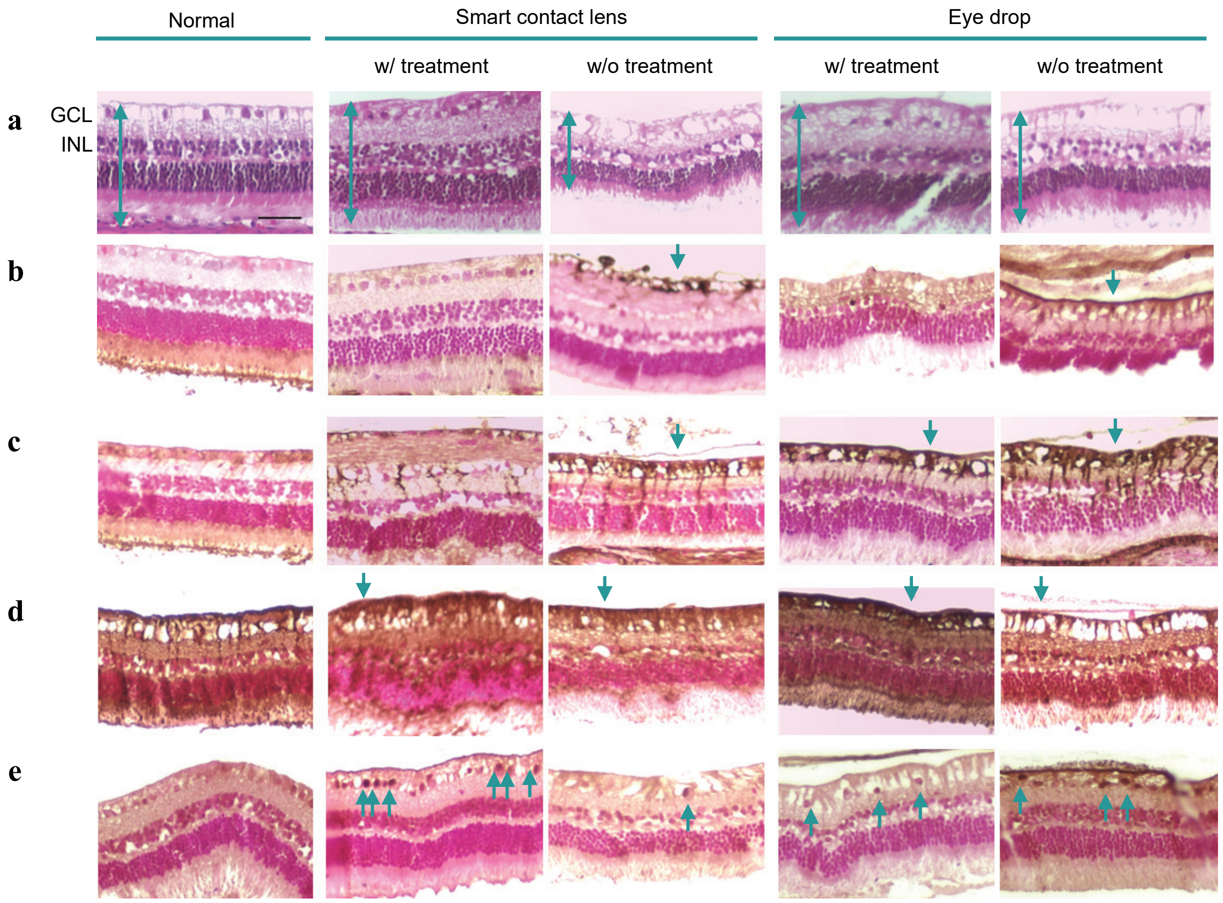

Supplementary Figure 18. In vivo therapeutic effect by theranostic smart contact lens and eye drop in glaucoma rabbits induced by methylcellulose. a, OM images for retinal histology. OM images for immunohistochemical analyses for **b**, GFAP, **c**, CD11b, **d**, BDNF and **e**, Brn3a. Arrows indicate the expression of each marker (scale bar, 100 μm).

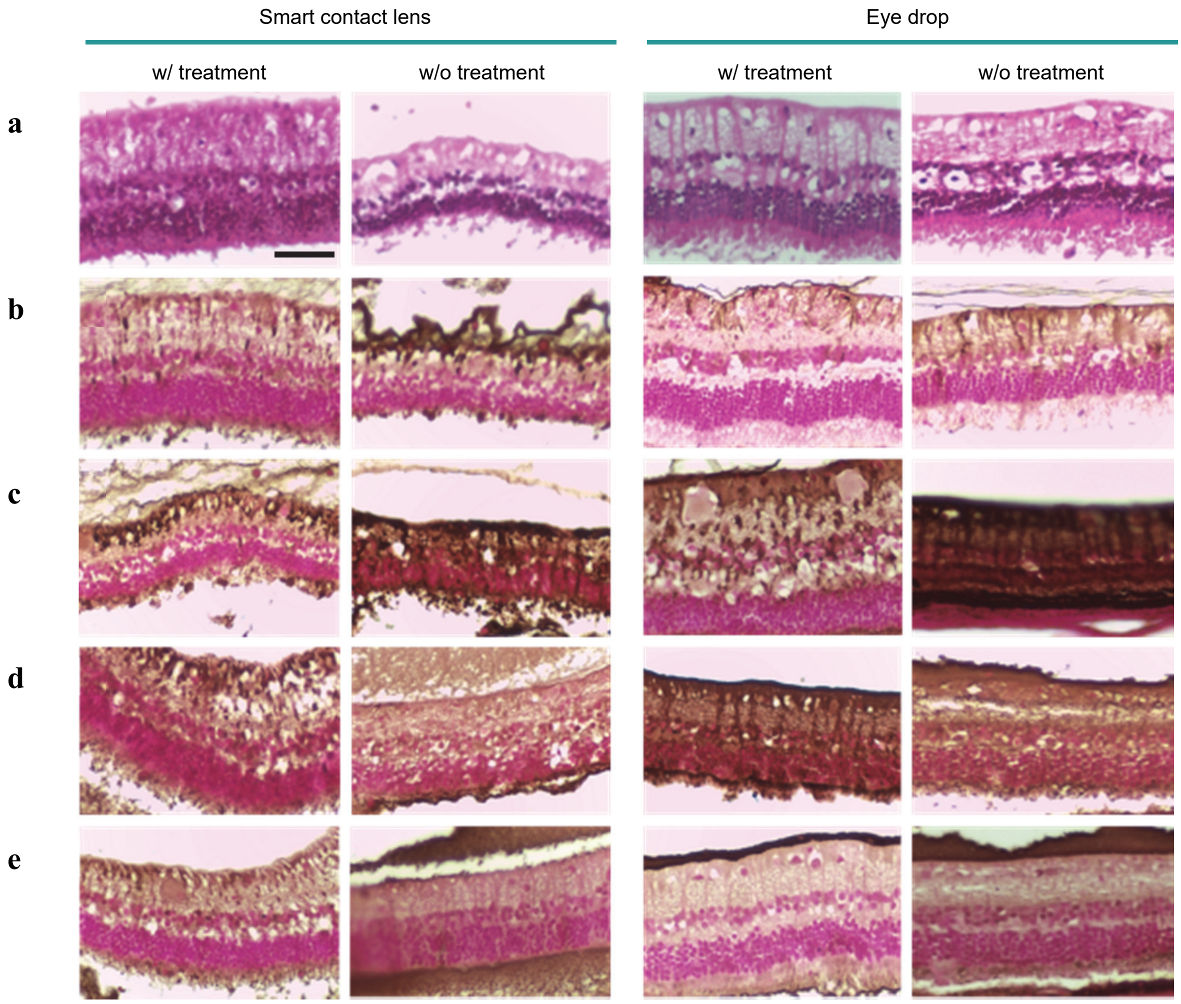

Supplementary Figure 19. In vivo therapeutic effect of theranostic smart contact lens and eye drops in glaucoma rabbits induced by α-chymotrypsin. a, OM images of retinal histology. OM images for immunohistochemical analyses with **b,** GFAP, **c,** CD11b, **d,** BDNF and **e,** Brn3a (scale bar, 100 μm).

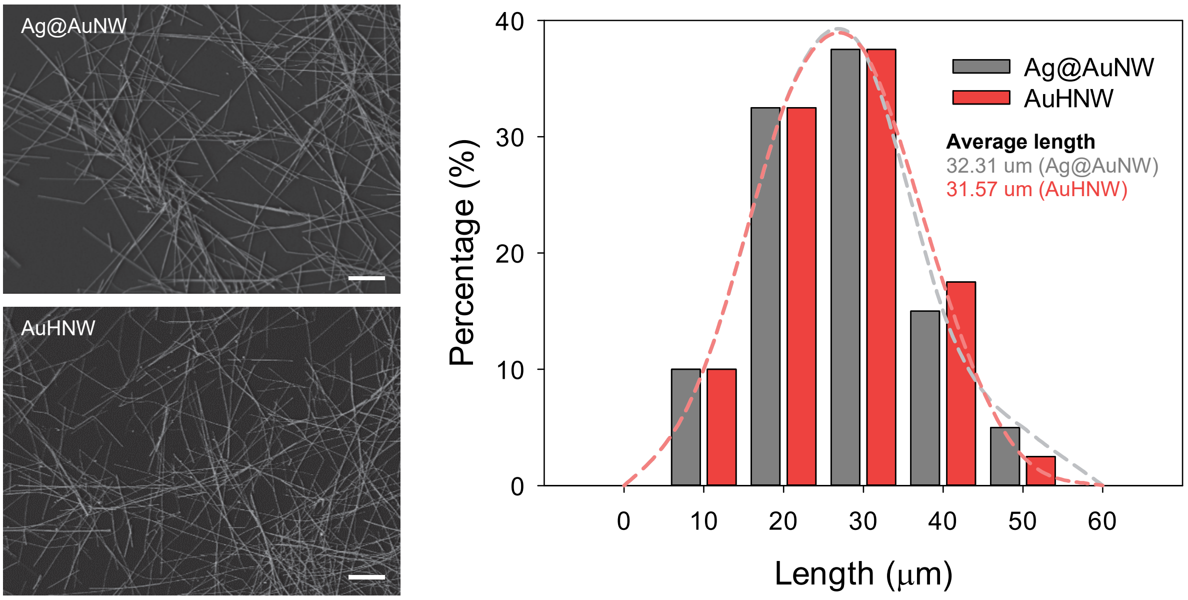

Supplementary Figure 20. The morphology and the size distribution of Ag@AuNWs and AuHNWs (scale bar, 10 μm).

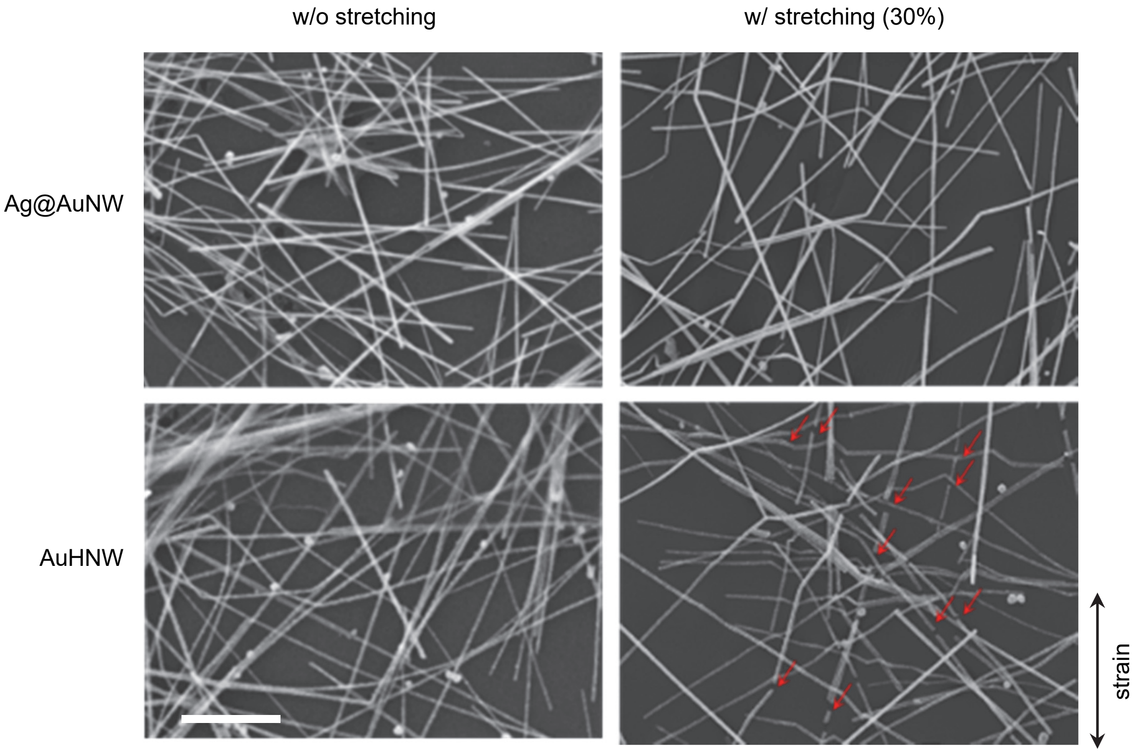

Supplementary Figure 21. SEM images of Ag@AuNW and AuHNW with and without applying strain. While there was no critical change in Ag@AuNW with mechanical stretching, cracks were observed through the strain direction in AuHNW with the applied strain. This fracture of AuHNW with strain resulted in the high sensitivity and reasonable stretchability of IOP sensor. Red arrows indicate the cracks of AuHNW (scale bar, 5 μm).

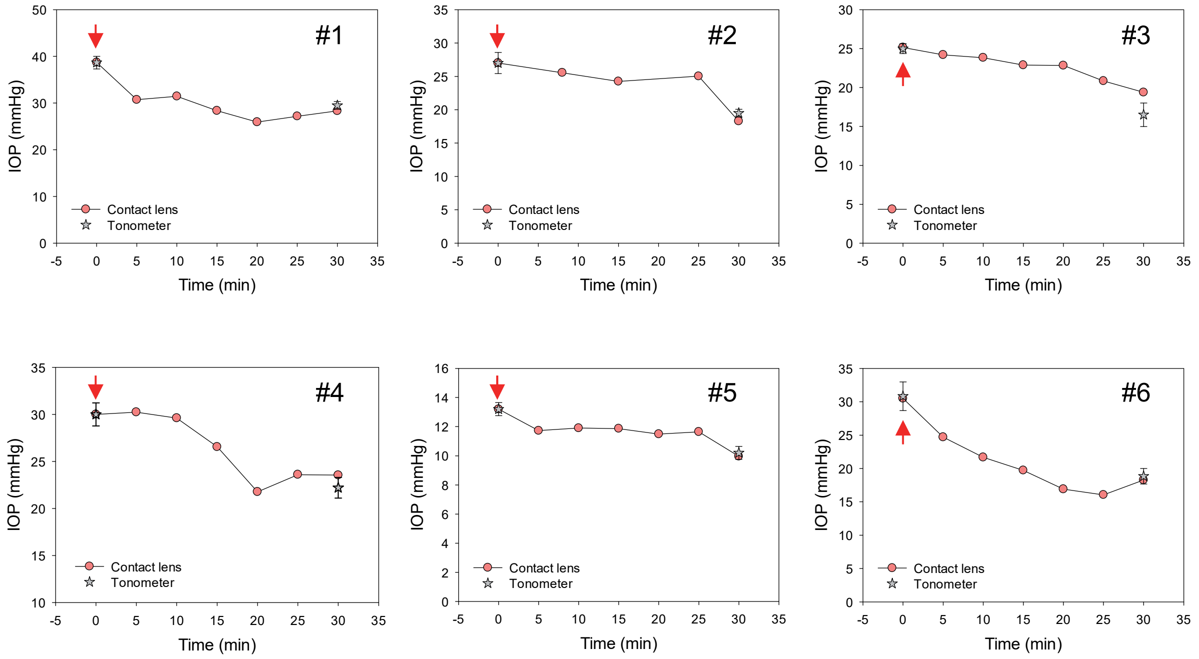

Supplementary Figure 22. The IOP change of glaucoma induced rabbits with drug treatment of theranostic smart contact lens. The IOP was successfully reduced by the treatment of drugs released from theranostic smart contact lens. The trend of IOP reduction was slightly different for each rabbit due to the different drug response or physiological condition. Each tonometer data is presented as a mean value ± SD (n = 6), except for #2 (n = 4), #4 and #5 (n = 5).

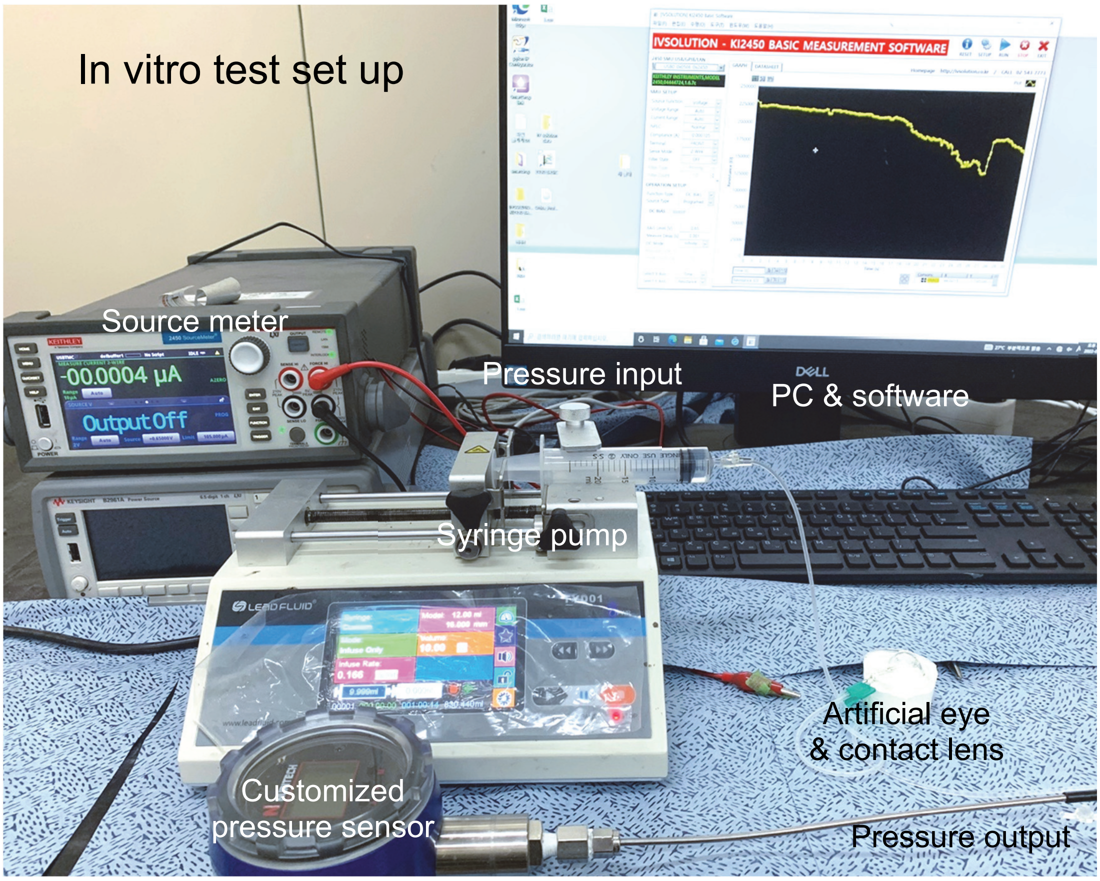

Supplementary Figure 23. The photograph for in vitro experimental set up.

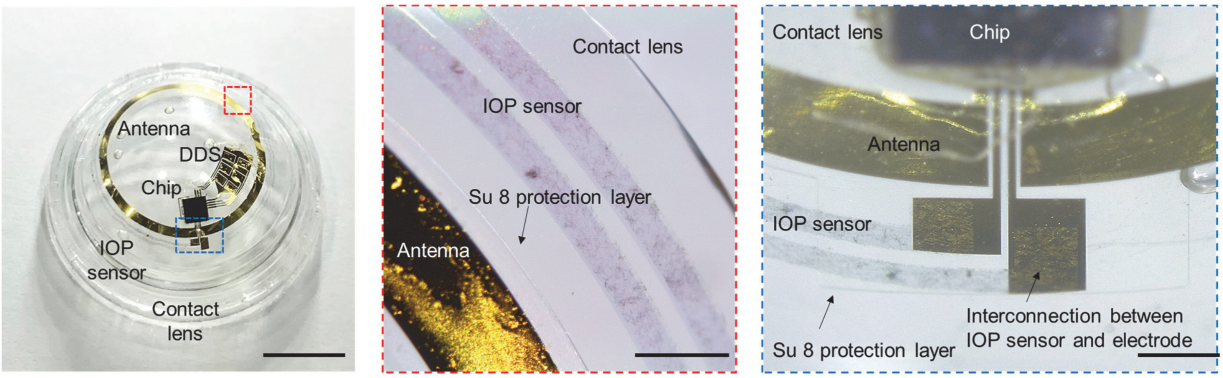

Supplementary Figure 24. Photographs of theranostic smart contact lens with photopatterned SU 8 layer for drug reservoirs and protection of antenna, chip and interconnection (scale bar, 5.5 mm for the left and 500 μm for the middle and right).
