# Supplementary material for: Wireless theranostic smart contact lens for monitoring and control of intraocular pressure in glaucoma
Source: Nat Commun. 2022 Nov 10;13:6801. doi: 10.1038/s41467-022-34597-8 (PMC9649789; doi:10.1038/s41467-022-34597-8)
Supplement: Supplementary file 3 — Reporting Summary [file 41467_2022_34597_MOESM3_ESM.pdf]

nature portfolio

Corresponding author(s): Sei Kwang Hahn

Last updated by author(s): Oct 23, 2022

Reporting Summary

Nature Portfolio wishes to improve the reproducibility of the work that we publish. This form provides structure for consistency and transparency in reporting. For further information on Nature Portfolio policies, see our Editorial Policies and the Editorial Policy Checklist.

Statistics

For all statistical analyses, confirm that the following items are present in the figure legend, table legend, main text, or Methods section.

n/a	Confirmed	
☐	☒	The exact sample size (n) for each experimental group/condition, given as a discrete number and unit of measurement
☐	☒	A statement on whether measurements were taken from distinct samples or whether the same sample was measured repeatedly
☐	☒	The statistical test(s) used AND whether they are one- or two-sided<br>*Only common tests should be described solely by name; describe more complex techniques in the Methods section.*
☒	☐	A description of all covariates tested
☒	☐	A description of any assumptions or corrections, such as tests of normality and adjustment for multiple comparisons
☐	☒	A full description of the statistical parameters including central tendency (e.g. means) or other basic estimates (e.g. regression coefficient) AND variation (e.g. standard deviation) or associated estimates of uncertainty (e.g. confidence intervals)
☐	☒	For null hypothesis testing, the test statistic (e.g. F, t, r) with confidence intervals, effect sizes, degrees of freedom and P value noted<br>*Give P values as exact values whenever suitable.*
☒	☐	For Bayesian analysis, information on the choice of priors and Markov chain Monte Carlo settings
☒	☐	For hierarchical and complex designs, identification of the appropriate level for tests and full reporting of outcomes
☒	☐	Estimates of effect sizes (e.g. Cohen's d, Pearson's r), indicating how they were calculated

Our web collection on statistics for biologists contains articles on many of the points above.

Software and code

Policy information about availability of computer code

Data collection	Custom algorithms are ancillary, but a full code is available in GitHub as a project name"NAT_SCL_2021." (https://github.com/cheonhoo-jeon/Nat_SCL_2021)<br>Commercial software programs of Xilinx ISE Design Suite (ver.14.7) and Java (ver.1.8.0_131) were used.<br>The LSK_v1.v is a Verilog code used in FPGA board to read data from RF receiver.<br>The guimake4.java is a java code to receive and plot data from the FPGA board on the computer.<br>The area fraction of each nanowire was analyze by the software program of Image J in this study.
Data analysis	Custom algorithms are ancillary, but a full code is available in GitHub as a project name"NAT_SCL_2021." (https://github.com/cheonhoo-jeon/Nat_SCL_2021)<br>Commercial software programs of Xilinx ISE Design Suite (ver.14.7) and Java (ver.1.8.0_131) were used.<br>The LSK_v1.v is a Verilog code used in FPGA board to read data from RF receiver.<br>The guimake4.java is a java code to receive and plot data from the FPGA board on the computer.<br>The area fraction of each nanowire was analyze by the software program of Image J in this study.

For manuscripts utilizing custom algorithms or software that are central to the research but not yet described in published literature, software must be made available to editors and reviewers. We strongly encourage code deposition in a community repository (e.g. GitHub). See the Nature Portfolio guidelines for submitting code & software for further information.

Data

Policy information about availability of data

All manuscripts must include a data availability statement. This statement should provide the following information, where applicable:

- Accession codes, unique identifiers, or web links for publicly available datasets
- A description of any restrictions on data availability
- For clinical datasets or third party data, please ensure that the statement adheres to our policy

The data generated in this study are provided in the Supplementary Information or Source Data file.

Human research participants

Policy information about studies involving human research participants and Sex and Gender in Research.

Reporting on sex and gender	This study did not involve human research.
Population characteristics	This study did not involve human research.
Recruitment	This study did not involve human research.
Ethics oversight	This study did not involve human research.

Note that full information on the approval of the study protocol must also be provided in the manuscript.

Field-specific reporting

Please select the one below that is the best fit for your research. If you are not sure, read the appropriate sections before making your selection.

☒ Life sciences ☐ Behavioural & social sciences ☐ Ecological, evolutionary & environmental sciences

For a reference copy of the document with all sections, see nature.com/documents/nr-reporting-summary-flat.pdf

Life sciences study design

All studies must disclose on these points even when the disclosure is negative.

Sample size	More than three samples or animals were used to achieve statistical significance depending on each experiment. The detailed sample size for each experiment is shown in all the figure legends.
Data exclusions	No data were excluded from the analyses.
Replication	All experiments were replicated and reproduced at least three times with independent experiments. We have specified error bars and we performed one-sided statistical analyses using on-way analysis of variance (ANOVA).
Randomization	We allocated samples and animals for complete randomization to avoid the biased experimental results.
Blinding	We carried out blind tests without knowing the tested samples for the experiment, data collection, and analysis.

Reporting for specific materials, systems and methods

We require information from authors about some types of materials, experimental systems and methods used in many studies. Here, indicate whether each material, system or method listed is relevant to your study. If you are not sure if a list item applies to your research, read the appropriate section before selecting a response.

Materials & experimental systems

n/a	Involved in the study
☐	☒ Antibodies
☐	☒ Eukaryotic cell lines
☒	☐ Palaeontology and archaeology
☐	☒ Animals and other organisms
☒	☐ Clinical data
☒	☐ Dual use research of concern

Methods

n/a	Involved in the study
☒	☐ ChIP-seq
☒	☐ Flow cytometry
☒	☐ MRI-based neuroimaging

Antibodies

Antibodies used	The antibodies for immunohistochemical analyses used here were GFAP (mouse, 1:1000 dilution, #sc-51908, SANTA CRUZ BIOTECHNOLOGY), Anti-CD11b antibody (rat, 1:1000 dilution, #ab8878, Abcam), Recombinant Anti-BDNF antibody (rabbit, 1:1000 dilution, #ab108319, Abcam), Recombinant Anti-Brn3a antibody (rabbit, 1:1000 dilution, #ab235230, Abcam).
Validation	Antibodies were validated according to manufacture's description

Eukaryotic cell lines

Policy information about cell lines and Sex and Gender in Research

Cell line source(s)	NIH 3T3 (mouse embryonic fibroblast) cell line was obtained from American Type Culture Collection (ATCC).
Authentication	Cell line was confirmed by morphology.
Mycoplasma contamination	All cell lines were tested negative for mycoplasma contamination.
Commonly misidentified lines (See ICLAC register)	no misidentified lines were used in this study.

Animals and other research organisms

Policy information about studies involving animals; ARRIVE guidelines recommended for reporting animal research, and Sex and Gender in Research

Laboratory animals	In order for animal tests, we used New Zealand White male rabbits (weighing 1.7 to 2.5 kg) with no signs of ocular inflammation.
Wild animals	This study did not involve wild animals.
Reporting on sex	This study did not involve reporting on sex.
Field-collected samples	This study did not involve samples collected from the field.
Ethics oversight	All animal experiments adhered to the ARVO Statement for the use of Animals in Ophthalmic and Vision Research with the approval by the Institutional Care and Use Committee (CRONEX-IACUC:20210005, CRONEX, Korea).

Note that full information on the approval of the study protocol must also be provided in the manuscript.
